# Pexidartinib improves physical functioning and stiffness in patients with tenosynovial giant cell tumor: results from the ENLIVEN randomized clinical trial

**DOI:** 10.1080/17453674.2021.1922161

**Published:** 2021-05-12

**Authors:** Michiel Van De Sande, William D Tap, Heather L Gelhorn, Xin Ye, Rebecca M Speck, Emanuela Palmerini, Silvia Stacchiotti, Jayesh Desai, Andrew J Wagner, Thierry Alcindor, Kristen Ganjoo, Javier Martín-Broto, Qiang Wang, Dale Shuster, Hans Gelderblom, John H Healey

**Affiliations:** a Department of Orthopedics, Leiden University Medical Center, Leiden, the Netherlands; b Department of Medicine, Memorial Sloan Kettering Cancer Center and Weill Cornell Medical College, New York, NY, USA; c Department of Patient-Centered Research, Evidera, Bethesda, MD, USA; d Department of Global Health Economics and Outcomes Research, Daiichi Sankyo Inc, Basking Ridge, NJ, USA; e Department of Experimental, Diagnostic, and Specialty Medicine, IRCCS Istituto Ortopedico Rizzoli, Bologna, Italy; f Department of Cancer Medicine, Fondazione IRCCS Istituto Nazionale dei Tumori, Milan, Italy; g Department of Medical Oncology, Peter MacCallum Cancer Centre, Melbourne, Victoria, Australia; h Department of Medical Oncology, Dana-Farber Cancer Institute, Boston, MA, USA; i Department of Medical Oncology, McGill University, Montreal, Quebec, Canada; j Department of Medical Oncology, Stanford Cancer Institute, Stanford, CA, USA; k Department of Medical Oncology, University Hospital Virgen del Rocio and Institute of Biomedicine of Sevilla (IBIS) (HUVR, CSIC, University of Sevilla), Seville, Spain; l Department of Biostatistics and Data Management, Daiichi Sankyo Inc, Basking Ridge, NJ, USA; m Department of Global Clinical Oncology Research and Development, Daiichi Sankyo Inc, Basking Ridge, NJ, USA; n Department of Medical Oncology, Leiden University Medical Center, Leiden, Netherlands; o Department of Orthopaedic Surgery, Memorial Sloan Kettering Cancer Center and Weill Cornell Medical College, New York, NY, USA

## Abstract

Background and purpose — The ENLIVEN trial showed that, after 25 weeks, pexidartinib statistically significantly reduced tumor size more than placebo in patients with symptomatic, advanced tenosynovial giant cell tumor (TGCT) for whom surgery was not recommended. Here, we detail the effect of pexidartinib on patient-reported physical function and stiffness in ENLIVEN.

Patients and methods — This was a planned analysis of patient-reported outcome data from ENLIVEN, a double-blinded, randomized phase 3 trial of adults with symptomatic, advanced TGCT treated with pexidartinib or placebo. Physical function was assessed using the Patient-Reported Outcomes Measurement Information System (PROMIS)-physical function (PF), and worst stiffness was assessed using a numerical rating scale (NRS). A mixed model for repeated measures was used to compare changes in PROMIS-PF and worst stiffness NRS scores from baseline to week 25 between treatment groups. Response rates for the PROMIS-PF and worst stiffness NRS at week 25 were calculated based on threshold estimates from reliable change index and anchor-based methods.

Results — Between baseline and week 25, greater improvements in physical function and stiffness were experienced by patients receiving pexidartinib than patients receiving placebo (change in PROMIS-PF = 4.1 [95% confidence interval (CI) 1.8–6.3] vs. –0.9 [CI −3.0 to 1.2]; change in worst stiffness NRS = –2.5 [CI −3.0 to −1.9] vs. –0.3 [CI −0.9 to 0.3]). Patients receiving pexidartinib had higher response rates than patients receiving placebo for meaningful improvements in physical function and stiffness. Improvements were sustained after 50 weeks of pexidartinib treatment.

Interpretation — Pexidartinib treatment provided sustained, meaningful improvements in physical function and stiffness for patients with symptomatic, advanced TGCT.

Tenosynovial giant cell tumor (TGCT) is a rare neoplasm that affects the joint synovia, bursae, and tendon sheaths (Gouin and Noailles 2017). Most patients experience pain, swelling, stiffness, instability, and reduced range of motion (Mastboom et al. 2018, Gelhorn et al. 2019). The current standard of care is surgical resection, but the tumors can be difficult to remove, frequently recur, and may require multiple surgeries or joint replacement (Ravi et al. 2011, van der Heijden et al. 2016). Therefore, non-surgical treatment options are needed.

Pexidartinib is an inhibitor of the colony-stimulating factor-1, KIT, and FLT3 receptor tyrosine kinases that was approved by the US Food and Drug Administration (FDA) for the treatment of adults with symptomatic TGCT associated with severe morbidity or functional limitations and not amenable to improvement with surgery (Lamb 2019). In ENLIVEN, a randomized, placebo-controlled phase 3 trial in 120 patients with symptomatic, advanced TGCT for whom surgery was not recommended, 39% of patients receiving pexidartinib and none of the patients receiving placebo at week 25 (p < 0.001) had a radiological response according to Response Evaluation Criteria in Solid Tumors, version 1.1 (RECIST 1.1). For patients receiving pexidartinib, 53% had a tumor response after about 2 years of treatment (Tap et al. 2019a).

In addition to assessing radiographic tumor response, ENLIVEN included patient-reported outcome (PRO) measures. This included items from the Patient-Reported Outcomes Measurement Information System (PROMIS)-physical function (PF) items list and a worst stiffness numerical rating scale (NRS), which were developed specifically for patients with TGCT (Gelhorn et al. 2019). Here, we detail the effects of pexidartinib on physical function and stiffness in ENLIVEN as measured by PROMIS-PF and worst stiffness NRS. We also examine the relationship between changes in these 2 PROs and changes in tumor size.

## Patients and methods

### Study design

This was a planned analysis of PRO data from ENLIVEN (NCT02371369), a 2-part, double-blinded, multinational phase 3 trial conducted at 39 hospitals and centers in the US, Canada, Europe, and Australia (Tap et al. 2019a). In part 1 of ENLIVEN (May 2015 to September 2016), eligible patients were randomized in a 1:1 ratio to receive pexidartinib for 24 weeks (1,000 mg/day for 2 weeks, then 800 mg/day for 22 weeks) or placebo for 24 weeks. Patients who completed part 1 could enter part 2 (ongoing), in which all patients received open-label pexidartinib.

Patients had to be ≥ 18 years of age, have histologically confirmed TGCT ≥ 2 cm as defined by RECIST 1.1 (Eisenhauer et al. 2009), be symptomatic, and have disease for which surgery could lead to worsening functional limitation or severe morbidity. Symptomatic disease was defined as a worst pain or worst stiffness score ≥ 4 on a scale of 0 (none) to 10 (worst imaginable) at any time during the week before the screening visit.

The primary endpoint in ENLIVEN was tumor response at week 25 based on central review of MRIs using RECIST 1.1, wherein size is measured as the sum of the tumor diameters (Eisenhauer et al. 2009). Tumor size was also measured by the tumor volume score (TVS), which corresponds to the percentage of the volume of the maximally distended synovial cavity or tendon sheath involved (Tap et al. 2015). The secondary endpoints in ENLIVEN were comparative analyses at week 25 of the (1) mean change from baseline in the range of motion of the affected joint; (2) the proportion of responders based on centrally evaluated MRI scans and TVS; (3) mean change from baseline in PROMIS-PF; (4) mean change from baseline in worst stiffness NRS; (5) proportion of responders based on the Brief Pain Inventory worst pain NRS and analgesic use by the Brief Pain Inventory-30 definition; and (6) duration of response based on RECIST and TVS (Tap et al. 2019a).

PROMIS-PF and worst stiffness NRS items were collected using an electronic handheld device.

### PROMIS-PF

Items from the 121-item PROMIS-PF item bank were selected to measure physical functioning in ENLIVEN. Rigorous methods were used to develop and validate items in the PROMIS-PF item bank (Rose et al. 2008, Bruce et al. 2009, Hays et al. 2013). The PROMIS-PF instrument used in ENLIVEN has been described (Gelhorn et al. 2016, 2019). The items in PROMIS-PF quantitatively measure the impact of TGCT on physical functioning (mobility, dexterity, axial, and complex activity function). 2 tumor location-specific PROMIS-PF forms were used: a 13-item bank customized to assess lower limb function among patients with lower extremity tumors (Gelhorn et al. 2019); and an 11-item bank customized to assess upper limb function among patients with upper extremity tumors. 9 of the PROMIS-PF items were overlapping (i.e., included in both lower and upper extremity scales), resulting in a total of 15 unique items. Each item has 5 response options ranging from “unable to do” to “able to do without any difficulty.” PROMIS-PF scores are expressed as T-scores, which are standardized to a mean of 50 and a standard deviation of 10, wherein a higher score represents better physical function. Content validity of PROMIS-PF has been demonstrated for patients with TGCT (Gelhorn et al. 2019).

### Worst stiffness NRS

Stiffness was evaluated using the worst stiffness NRS, a single-item self-administered questionnaire that assessed worst stiffness at site of the tumor in the last 24 hours (Gelhorn et al. 2016, 2019). The item has a response scale of 0 to 10, where 0 is “no stiffness” and 10 is “stiffness as bad as you can imagine.” A daily stiffness score was reported, and each patient’s weekly stiffness score was calculated as the average of completed records. A minimum of 4 out of 7 days of data was necessary to compute a weekly mean. Content validity of the worst stiffness NRS has been demonstrated for patients with TGCT (Gelhorn et al. 2019).

### Statistics

Statistical analyses were performed in the intent-to-treat population using SAS version 9.4 (SAS Institute, Cary, NC, USA). Subjects were analyzed in the treatment group to which they were randomized.

PROMIS-PF scores were derived using item-response theory parameters for each item (PROMIS 2020). Pattern-based scores for the custom PROMIS short forms were estimated using PROC IRT in SAS version 9.4 (SAS Institute, Cary, NC, USA).

As planned analyses prior to database lock and unblinding, changes in PROMIS-PF and worst stiffness NRS scores from baseline to week 25 were compared between treatment groups using a mixed model for repeated measures. The models specified the change in scores from baseline as the dependent variable and treatment group, time point, treatment group-by-time interaction, stratification factor of US sites versus non-US sites, the baseline value of the corresponding endpoint, and the baseline-by-time interaction as independent variables. Changes in PROMIS-PF and worst stiffness NRS scores from baseline to week 25 were analyzed using a hierarchical testing strategy to address multiplicity issues (Alosh et al. 2014, Tap et al. 2019a). A p-value of < 0.05 was considered statistically significant.

Due to substantial missing post-baseline data, post hoc sensitivity analyses were performed to assess the robustness and consistency of the mixed-model repeated-measure analysis results, which included unconditional jump to reference (Carpenter et al. 2013) and delta adjustment tipping point (Mehrotra et al. 2017) analyses, both without the missing-at-random assumption. For the unconditional jump to reference analysis, missing data were imputed as follows: once a participant discontinued pexidartinib treatment, all of the attained treatment benefits were assumed to disappear, and the imputations were modelled as under placebo treatment. The delta adjustment tipping point analysis imputed missing data on the pexidartinib treatment arm by first imputing a value based on the missing-at-random assumption and then imposing a penalty of size delta to discontinued patients and a penalty of size half of delta to patients who completed part 1 of the study. The penalty was applied sequentially, and thus the 2nd missing value of a patient was assigned another delta penalty when the missing value was imputed. Missing data for patients on the placebo treatment arm were not assigned any penalty when being imputed or following missing-at-random assumption. The tipping point was determined as the point at which statistical significance was lost.

The proportion of patients achieving meaningful within-patient change thresholds for PROMIS-PF and worst stiffness NRS at week 25 between treatments was compared by Fisher’s exact test and displayed via empirical cumulative distribution function curves. If assessments were missing or sufficient data were not available to calculate the endpoint, patients were considered not to have achieved the threshold. The reliable change index (RCI), defined as 1.9_*_SQR (2)_*_SEM = 2.77_*_SEM, where SEM = standard error of measurement = SD (SQR (1 – reliability)) (Hays and Peipert 2018) and anchor-based methods (US Food and Drug Administration 2009) were used to estimate meaningful within-patient change thresholds. For PROMIS-PF in the TGCT population, the RCI yields a value of 6.84, and a previously published anchor-based estimate yielded a value of ≥ 3-point increase. For worst stiffness NRS in the TGCT population, the RCI value is 1.3, while the previously published anchor-based estimate is 1 (Tap et al. 2019b).

For patients who continued treatment during the open-label extension (part 2) for at least 50 weeks, mean changes from baseline in PROMIS-PF and worst stiffness NRS after 25 and 50 weeks of treatment were reported.

Correlations between changes from baseline to week 25 in PRO scores (PROMIS-PF and worst stiffness NRS) and changes in tumor size (sum of diameters and TVS) were examined using Pearson’s correlation (r) for all evaluable patients.

Estimates are presented with their 95% confidence intervals (CI).

### Ethics, funding, data sharing, and potential conflicts of interest

ENLIVEN was approved by an institutional review board at each participating center and conducted in accordance with the Declaration of Helsinki and International Council on Harmonisation guidelines on Good Clinical Practice. All patients provided written informed consent.

This study was supported by Daiichi Sankyo. De-identified individual participant data and supporting clinical study documents are available on request, depending on circumstances, at https://vivli.org.

MvdS, EP, SS, JD, AJW, and HG received institutional grants from Daiichi Sankyo unrelated to the submitted work. WDT, SS, JD, AJW received personal fees from Daiichi Sankyo unrelated to the submitted work. WDT has patents pending on a companion diagnostic for CDK4 inhibitors and a treatment for metastatic sarcoma and is on scientific advisory boards for companies other than Daiichi Sankyo. MvdS, EP, SS, JD, AJW, TA, JMB, and HG received institutional grants from pharmaceutical companies other than Daiichi Sankyo unrelated to the submitted work. WDT, EP, SS, JD, AJW, TA, and JMB received personal fees from pharmaceutical companies other than Daiichi Sankyo unrelated to the submitted work. XY, QW, and DS are employees of Daiichi Sankyo. HLG and RMS are employees of Evidera, which received funding from Daiichi Sankyo. JHH is a paid consultant of Daiichi Sankyo.

## Results

### Patients

The analysis included 120 patients with symptomatic TGCT randomized and treated in ENLIVEN (59 to placebo, 61 to pexidartinib) (Figure 1, see Supplementary data). The patients had a mean age of 45 (SD 13) years, 59% were female, and 88% were white. Most TGCTs (92%) were in the lower extremities, most often in the knee (61%) and ankle (18%). Nearly all patients (98%) indicated that their tumor limited their physical function. 48 patients receiving placebo and 52 receiving pexidartinib completed part 1 of the study. In the open-label extension (part 2), 30 patients receiving placebo switched to pexidartinib and 48 patients who started on pexidartinib continued receiving it. Further details of patient characteristics have been published (Tap et al. 2019a).

### Effect of pexidartinib on physical function and stiffness at the end of part 1 (week 25)

At week 25, patients receiving pexidartinib had greater improvements in PROMIS-PF and worst stiffness NRS than patients receiving placebo. The mean change in PROMIS-PF was 4.1 (CI 1.8–6.3) in patients receiving pexidartinib and −0.9 (CI −3.0 to 1.2) in patients receiving placebo, and the mean change in worst stiffness NRS was −2.5 (CI −3.0 to 1.9) in patients receiving pexidartinib and −0.3 (CI −0.9 to 0.3) in patients receiving placebo (Table 1).

**Table 1.** Changes in PROMIS-PF and worst stiffness NRS between baseline and the end of part 1 (week 25)

	Placebo	Pexidartinib	
Outcome	(n = 59)	(n = 61)	p-value
PROMIS-PF score **^a^**			
Baseline, n	57	60	
Week 25, n	32	39	
Change between baseline and week 25, LS mean (95% CI)			
Mixed-effect model repeated measures model **^b^**	−0.9 (–3.0 to 1.2)	4.1 (1.8–6.3)	0.002
Unconditional jump to reference model **^c^**	−0.9 (–3.0 to 1.3)	3.5 (1.3–5.8)	0.005
Response at week 25 **^d,e^**, n (%)	3 (5)	18 (30)	< 0.001
Worst stiffness NRS score			
Baseline, n	58	59	
Week 25, n	36	34	
Change between baseline and week 25, LS mean (95% CI)			
Mixed-effect model repeated measures model **^b^**	−0.3 (–0.9 to 0.3)	−2.5 (–3.0 to –1.9)	< 0.001
Unconditional jump to reference model **^c^**	−0.3 (–0.9 to 0.4)	−2.2 (–2.8 to –1.6)	< 0.001
Response at week 25 **^e,f,^** n (%)	11 (19)	24 (39)	0.02

Abbreviations: CI, confidence interval; LS, least squares; NRS, numerical rating scale;

PROMIS-PF, Patient-Reported Outcomes Measurement Information System-physical function items.

**^a^**Physical function was assessed daily using 15 items from the Patient-Reported Outcomes Measurement Information System (PROMIS)-physical function (PF) item bank, and a weekly average was calculated at baseline and at week 25.

**^b^**Primary assessment. The model specified the change in scores from baseline as the dependent variable and treatment group, time point, treatment group-by-time interaction, stratification factor of US sites versus non-US sites, the baseline value of the corresponding endpoint, and the baseline-by-time interaction as independent variables.

**^c^**Post hoc sensitivity analysis. Missing data were imputed by the missing not-at-random assumption.

**^d^**Increase of ≥ 3 points from baseline.

**^e^**Compared by 2-sided Fisher’s exact test.

**^f^**Decrease of ≥ 1 point from baseline .

Week-25 PRO data were missing for as many as 46% of the patients in each treatment group due to discontinuations, patient non-compliance, and technical reasons (Figure 1, see Supplementary data). Therefore, to confirm the robustness and consistency of the findings, post hoc sensitivity analyses were conducted. Changes from baseline to week 25 remained similar using an unconditional jump to reference method: the mean change in PROMIS-PF was 3.5 (CI 1.3–5.8) in patients receiving pexidartinib and −0.9 (CI −3.0 to 1.3) in patients receiving placebo, and the mean change in worst stiffness NRS was −2.2 (CI −2.8 to −1.6) in patients receiving pexidartinib and −0.3 (CI −0.9 to 0.4) in patients receiving placebo (see Table 1). Tipping point analysis showed that the required delta for loss of statistical significance was −3.6 for PROMIS-PF and 2.5 for worst stiffness NRS, which are greater than the estimated treatment effects based on the observed data.

The empirical cumulative distribution function curves for PROMIS-PF and worst stiffness NRS depicting the proportion of participants in each treatment group reporting each level of change are shown in Figures 2 and 3 (see Supplementary data). These figures show large and consistent differences between the treatment and placebo groups. At both the RCI and anchor-based thresholds, at week 25, a greater proportion of patients receiving pexidartinib than placebo achieved meaningful change in physical function (RCI, n = 10/61 vs. 2/59, p = 0.02; anchor-based: n = 18/61 vs. 3/59, p < 0.001) and stiffness (RCI, n = 24/61 vs. 11/59, p = 0.01; anchor-based: n = 24/61 vs. 11/59, p = 0.02).

### Effect of pexidartinib on physical function and stiffness during the open-label extension (part 2)

In patients who started on pexidartinib and continued to receive it open label during part 2 (n = 48), improvements were sustained in both PROMIS-PF (change from baseline = 3.6 [CI 2.0–5.2] at week 25 and 4.7 [CI 3.0–6.5] at week 50) and worst stiffness NRS (change from baseline = −2.7 [CI −3.4 to −1.9] at week 25 and −3.5 [CI −4.3 to −2.6] at week 50) (Table 2). Improvements were also sustained in patients who started on placebo and switched to pexidartinib (n = 30) as measured by both PROMIS-PF (change from baseline = 4.9 [CI 1.5–8.3] after 25 weeks on pexidartinib and 7.6 [CI 4.0–11.3] after 50 weeks on pexidartinib) and worst stiffness NRS (change = −3.0 [CI −4.5–1.5] after 25 weeks on pexidartinib and −2.2 [CI −4.2 to −0.2] after 50 weeks on pexidartinib). Overall, for patients included in the open-label extension, PROMIS-PF increased by 4.0 (CI 2.5–5.4) after 25 weeks on pexidartinib and by 5.8 (CI 4.1–7.5) after 50 weeks, while worst stiffness NRS changed by −2.8 (CI −3.5 to −2.1) after 25 weeks on pexidartinib and by −3.1 (CI −3.9 to −2.3) after 50 weeks.

**Table 2.** Changes in PROMIS-PF and worst stiffness NRS during the open-label extension (part 2)

Outcome	Received placebo during part 1, switched to pexidartinib during part 2 (n = 30)	Received pexidartinib during parts 1 and 2 (n = 61)	All patients treated with pexidartinib (n = 91)
PROMIS-PF score **^a^**			
Change after 25 weeks			
on pexidartinib, n	16	38	54
mean (95% CI)	4.9 (1.5 to 8.3)	3.6 (2.0 to 5.2)	4.0 (2.5 to 5.4)
Change after 50 weeks			
on pexidartinib, n	14	25	39
mean (95% CI)	7.6 (4.0 to 11.3)	4.7 (3.0 to 6.5)	5.8 (4.1 to 7.5)
Worst stiffness NRS score			
Change after 25 weeks			
on pexidartinib, n	18	33	51
mean (95% CI)	−3.0 (−4.5, to 1.5)	−2.7 (−3.4 to −1.9)	−2.8 (−3.5 to −2.1)
Change after 50 weeks			
on pexidartinib, n	10	22	32
mean (95% CI)	−2.2 (−4.2 to −0.2)	−3.5 (−4.3 to −2.6)	−3.1 (−3.9 to − 2.3)

Abbreviations: CI, confidence interval; NRS, numeric rating scale; PROMIS-PF, Patient-Reported Outcomes Measurement Information System-physical function items.

**^a^**Physical function was assessed daily using 15 items from the Patient-Reported Outcomes Measurement Information System (PROMIS)-physical function (PF) item bank, and a weekly average was calculated at baseline and at week 25.

### Correlation between changes in tumor size and PROs in part 1

**Figure 4.**
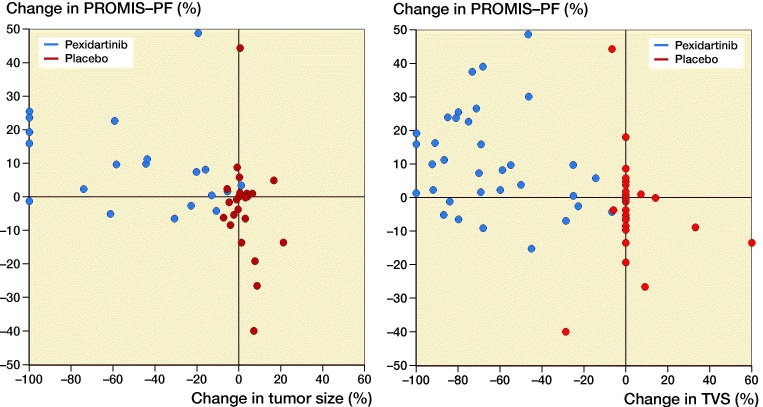
Correlation between change in physical function and change in tumor size (sum of diameters of target lesions) and tumor volume score (TVS) in adults with symptomatic, advanced tenosynovial giant cell tumor treated with pexidartinib or placebo in ENLIVEN. In part 1 of ENLIVEN, adult patients with symptomatic, advanced tenosynovial giant cell tumor were randomized to treatment with pexidartinib (1,000 mg/day for 2 weeks, then 800 mg/day for 22 weeks) or placebo for 24 weeks. Physical function was assessed daily using 15 items from the Patient-Reported Outcomes Measurement Information System (PROMIS)-physical function (PF) item bank, and a weekly average was calculated at baseline and at week 25. The figure shows that improvement between baseline and week 25 in PROMIS-PF correlated with the reduction in tumor size during the same period when measured by either RECIST 1.1 (Pearson’s r = −0.49, p = 0.0008; panel A) or tumor volume score (TVS; Pearson’s r = −0.34, p = 0.006; panel B).

**Figure 5.**
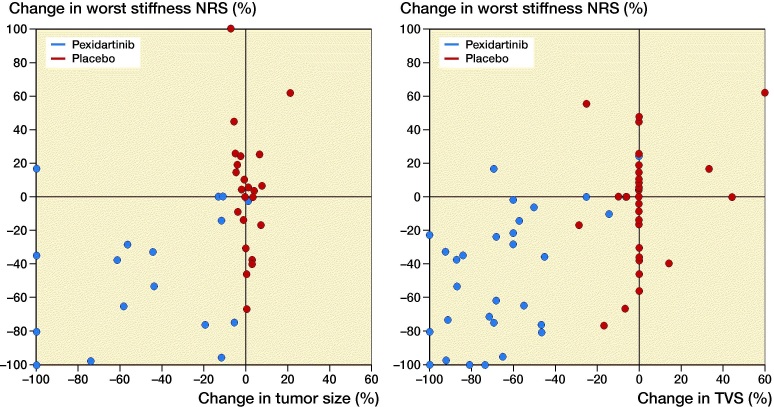
Correlation between change in worst stiffness and change in tumor size (sum of diameters of target lesions) and tumor volume score (TVS) in adults with symptomatic, advanced tenosynovial giant cell tumor treated with pexidartinib or placebo in ENLIVEN. In part 1 of ENLIVEN, adult patients with symptomatic, advanced tenosynovial giant cell tumor were randomized to treatment with pexidartinib (1,000 mg/day for 2 weeks, then 800 mg/day for 22 weeks) or placebo for 24 weeks. Worst stiffness was assessed daily using a numerical rating scale (NRS), and a weekly average was calculated. The figure shows that improvement between baseline and week 25 in worst stiffness NRS correlated with the reduction in tumor size during the same period when measured by either RECIST 1.1 (Pearson’s r = 0.53, p = 0.0004; panel A) or tumor volume score (TVS; Pearson’s r = 0.43, p = 0.0003; panel B).

Improvement from baseline in PROMIS-PF after 25 weeks of treatment correlated with a reduction of tumor size as measured by RECIST 1.1 (r = −0.5, p = 0.0008; Figure 4A) and TVS (r = −0.3, p = 0.006; Figure 4B). Improvement from baseline in worst stiffness NRS after 25 weeks of treatment also correlated with a reduction of tumor size as measured by RECIST 1.1 (r = 0.5, p < 0.001; Figure 5A) and TVS (r = 0.4, p < 0.001; Figure 5B). The correlation plots revealed that for patients whose physical function or stiffness worsened, the extent of worsening was, in most cases, less in patients receiving pexidartinib than in patients receiving placebo. The plots also revealed that, although some patients receiving pexidartinib continued to have some worsening physical function or stiffness, only a single patient had a small increase in the sum of diameters of target lesions (but no increase in TVS).

## Discussion

This analysis of data from ENLIVEN confirmed that treatment with pexidartinib produced sustained, meaningful improvements in physical function and stiffness. These improvements corresponded with reductions in tumor size reported previously (Tap et al. 2019a). The results also showed that the PROs, PROMIS-PF, and worst stiffness NRS can be used in TGCT clinical trials to assess outcomes from the patient perspective.

PROMIS-PF and worst stiffness NRS were included in ENLIVEN to confirm that the decreases in tumor size reflect changes in the tumors that are meaningful to patients. These 2 PROs were adapted specifically for patients with TGCT through a process of a targeted literature review, clinical expert interviews, and cognitive debriefing to confirm that the instructions, questions, and response options were relevant to and understood by patients. The content validity of the measures of these 2 PROs for patients with TGCT has previously been described (Gelhorn et al. 2019). A previous psychometric study demonstrated that, for these patients, PROMIS-PF has acceptable internal consistency reliability and that the 2 instruments have good test–retest reliability, have adequate convergent validity with other PRO measures, can differentiate between known groups, and can detect change over time (Speck et al. 2020).

The improvements in PROMIS-PF and worst stiffness NRS correlated moderately with decreases in tumor size. This moderate correlation was because some patients continued to experience reduced physical function or stiffness despite reduced tumor size. This may be due to the residual tumor affecting surrounding tissues or to continuing synovitis, underlying degenerative joint disease, and possibly sequelae from earlier surgeries.

Improvements in PROMIS-PF were greater in patients who started on placebo and switched to pexidartinib than patients who started on pexidartinib and continued to receive it. This finding may be due to the relatively small sample size in this analysis and individual differences in physical function improvement. Because part 2 of ENLIVEN was open label, patients who switched over from placebo may have expected a “treatment effect,” which could have biased the results.

A strength of this analysis is that the PROs were developed specifically for patients with TGCT and, according to FDA guidance, have acceptable psychometric properties (Gelhorn et al. 2019). Other PROs, including the 36-Item Short-Form Health Survey, visual analogue scale for pain, and Western Ontario and McMaster Universities Osteoarthritis Index, have been used to examine the effect of surgery on quality of life and joint function, but none of them are specific to TGCT (Verspoor et al. 2019). Thus, the current study provides valid, robust data on how patients with TGCT perceive the effects of treatment with pexidartinib. Another strength of this analysis is that it relied on the results of a randomized, placebo-controlled clinical trial, which provided robust, prospective data and controlled for patient bias.

Nonetheless, some limitations should be considered when interpreting the results. Most of all, substantial post-baseline data were missing for PROMIS-PF and worst stiffness NRS due to discontinuations, patient non-compliance, and technical issues with the electronic data-collection device. This included 8 patients who discontinued pexidartinib due to hepatic adverse events, including 4 cases of mixed or cholestatic hepatotoxicity, which led to the US FDA establishing a risk evaluation and mitigation strategy (REMS) program. Sensitivity analyses conducted to address the potential of informative missing data confirmed that the differences in scores between placebo and pexidartinib were statistically significant, although estimates of treatment effect size could have been affected. In addition, although a conservative approach was taken to handling missing data by assuming that all patients with missing data were non-responders, response rates for placebo and pexidartinib remained statistically significantly different. Another potential limitation of this study is that follow-up data on symptoms for patients who discontinued the study were not collected, so we could not determine the duration of benefits of pexidartinib after treatment was discontinued. Finally, we did not determine whether the results differed according to tumor location. Nonetheless, the results support the conclusion that pexidartinib durably improves physical function and stiffness from the patient perspective.

In conclusion, this analysis demonstrated the benefit of pexidartinib in patients with symptomatic TGCT for whom surgery, if possible, would be associated with severe morbidity or functional limitation. This benefit must be carefully balanced against the risk of severe liver and other toxicities.

## Supplementary Material

Supplemental Material

## Supplementary data

Figures 1–3 are available as supplementary data in the online version of this article, http://dx.doi.org/10.1080/17453674.2021. 1922161
