# Supplementary material for: Pexidartinib improves physical functioning and stiffness in patients with tenosynovial giant cell tumor: results from the ENLIVEN randomized clinical trial
Source: Acta Orthop. 2021 May 12;92(4):493–9. doi: 10.1080/17453674.2021.1922161 (PMC8382018; doi:10.1080/17453674.2021.1922161)
Supplement: Supplemental Material [file IORT_A_1922161_SM4562.pdf]

Supplementary data

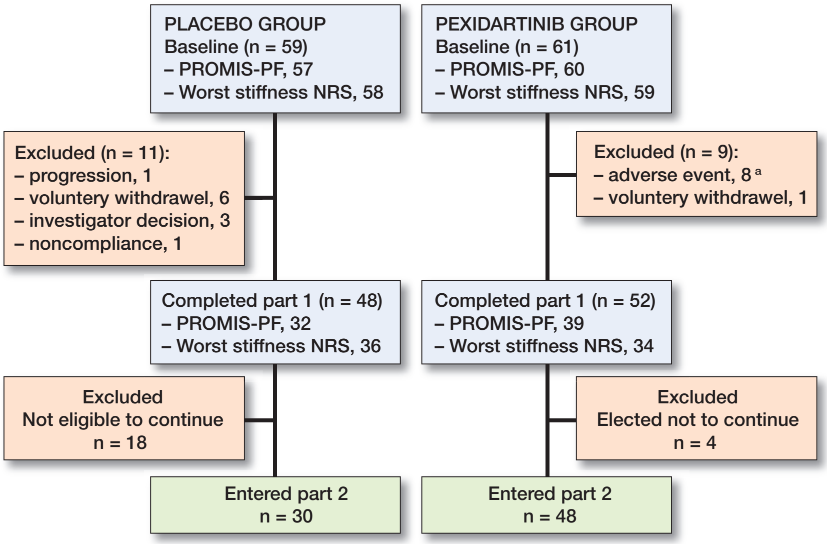

Figure 1. Disposition flowchart of patients with symptomatic tenosynovial giant cell tumor randomized to treatment with pexidartinib or placebo in ENLIVEN. Abbreviations: NRS, numerical rating scale; PROMIS-PF, Patient-Reported Outcomes Measurement Information System-physical function.
[a] 7 of the 8 adverse events leading to study discontinuation were liver-related.

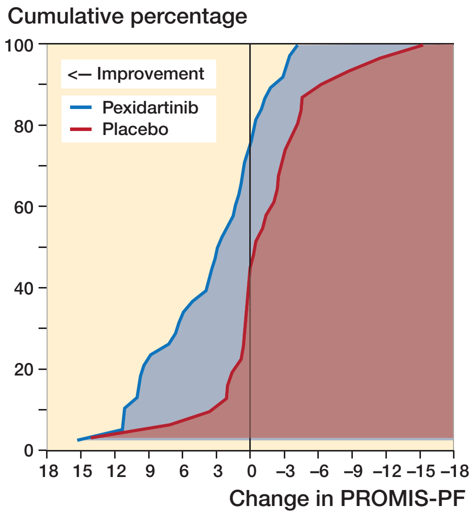

Figure 2. Empirical cumulative distribution function curve of change in PROMIS-PF from baseline to week 25 in patients with symptomatic tenosynovial giant cell tumor randomized to treatment with pexidartinib or placebo in ENLIVEN. Abbreviations: PROMIS-PF, Patient-Reported Outcomes Measurement Information System–physical function.

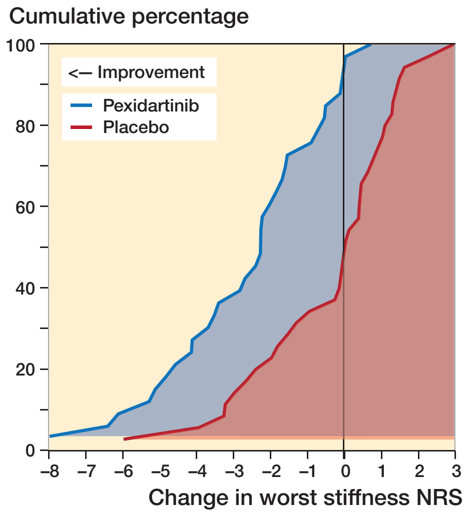

Figure 3. Empirical cumulative distribution function curve of change in worst stiffness NRS from baseline to week 25 in patients with symptomatic tenosynovial giant cell tumor randomized to treatment with pexidartinib or placebo in ENLIVEN. Abbreviation: NRS, numerical rating scale.
